# An ancient defense mechanism: Conservation of gasdermin-mediated pyroptosis

**DOI:** 10.1371/journal.pbio.3002103

**Published:** 2023-05-04

**Authors:** Chengliang Wang, Jianbin Ruan

**Affiliations:** Department of Immunology, School of Medicine, University of Connecticut Health Center, Farmington, Connecticut, United States of America

## Abstract

Gasdermin (GSDM) is a family of pore-forming proteins involved in various cellular processes such as cell death and inflammation. This Primer explores a PLOS Biology study of the evolutionary history of GSDMs across metazoan species, including Amphioxus, highlighting the conservation and divergence of GSDME in inducing pyroptosis in response to infection.

## Teaser

The gasdermins are a family of pore-forming proteins involved in various cellular processes such as cell death and inflammation. A new study in *PLOS Biology* explores the evolutionary history of gasdermins across metazoans, highlighting the conservation and divergence of gasdermin E.

Pyroptosis is a highly pro-inflammatory form of lytic cell death that serves as the first line of host defense by eliminating intracellular replication niches and enhancing the host’s defensive responses. The gasdermins, a family of pore-forming proteins, are executioners of pyroptosis. Gasdermins have a well-conserved domain architecture that contains an N-terminal pore-forming domain, a C-terminal autoinhibitory domain, and a highly divergent interdomain linker, and share a unified mechanism of autoinhibition and activation [1,2]. In response to microbial infections and other danger signals, activated caspases, granzymes, and bacterial proteases can recognize and cleave the interdomain linker in gasdermins, unleashing the pyroptotic N-terminal fragments that form pores in cell membranes and trigger pyroptosis [3–7]. Gasdermins have been widely studied in recent years, and emerging roles in autoimmune and inflammatory diseases, deafness, and cancers have highlighted their strong potential as therapeutic targets.

*GSDM* (the gene encoding gasdermin) and *GSDM*-like genes are evolutionarily ancient and have been identified across a wide range of animal and nonanimal species, including fungi and bacteria. In humans, the *GSDM* gene family consists of 6 paralogs, including *GSDMA*, *GSDMB*, *GSDMC*, *GSDMD*, *GSDME* (also known as *DFNA5*), and *DFNB59* (also known as *PJVK*, encoding pejvakin). Previous studies on gasdermins mostly focused on their functional roles in the immune response, and their origin and evolution have not been well studied. In this issue of *PLOS Biology*, Wang and colleagues investigated the evolutionary history of *GSDM* genes across metazoan species using bioinformatic methods [8]. Consistent with another recent study [9], Wang and colleagues found that *GSDME* is the most ancient *GSDM* gene and that the evolution of gasdermins can be divided into 2 clades: the gasdermin A/B/C/D clade, which is specific to jawed vertebrates, and the gasdermin E/pejvakin clade, which is widely distributed from cnidaria to mammals [8]. Within the gasdermin E/pejvakin clade, *PJVK* was a duplication from *GSDME* but lost the last 3 exons that encode the C-terminal autoinhibitory domain in early vertebrates. In the gasdermin A/B/C/D clade, *GSDMA* and *GSDMB* arose about 400 million years ago as a duplication of *GSDME* in cartilaginous fish. *GSDMD* emerged by duplication from *GSDMB* and then duplicated to form *GSDMC* in early mammals. Moreover, analysis of the intron phases of *GSDM* genes indicated that the evolution of the gasdermin family is dynamic and influenced by the coevolution of host and pathogen.

Gasdermin-mediated pyroptosis can be activated through a variety of signaling pathways. Canonical and noncanonical inflammasome activation induces gasdermin D–mediated pyroptosis, promoting protective immune responses [3,4]. Alternatively, granzyme A, delivered by cytotoxic lymphocytes, cleaves gasdermin B, triggering pyroptosis in target cells and promoting tumor clearance in mice [7], and gasdermin A is cleaved by group A *Streptococcus* cysteine protease SpeB in response to infection [5]. Gasdermin E was first known to have a role in antitumor immunity by inducing pyroptosis in certain cancer cells through cleavage by caspase-3 [6]. A later study in teleosts demonstrated that gasdermin E–mediated pyroptosis is also important for immune defense against bacterial infection [10]. However, pyroptosis in invertebrates, which only have ancestral gasdermin E, is currently unclear.

**Fig 1:**
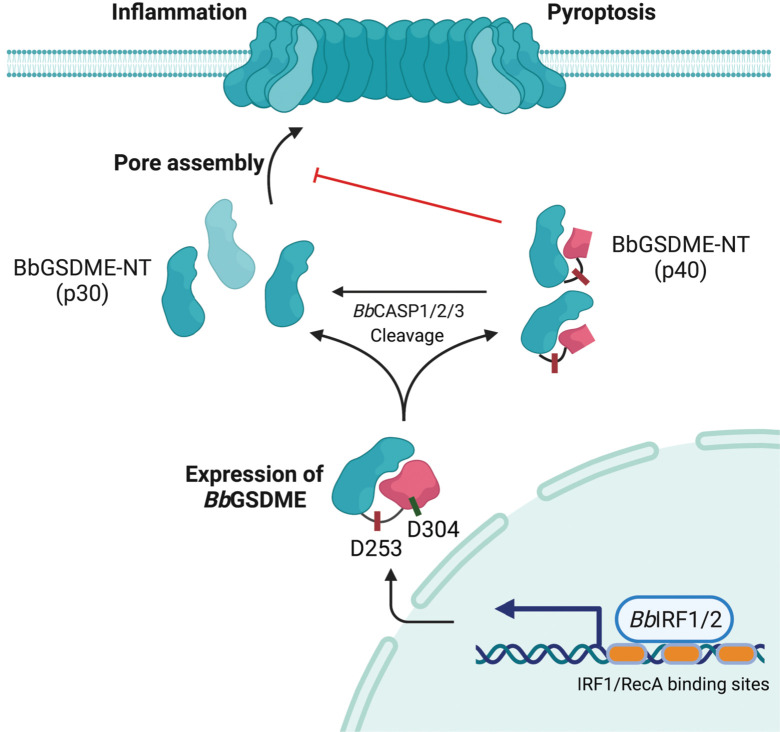
Regulation and activation of gasdermin E in amphioxus. In amphioxus, transcription factors IRF1 and IRF8 promote the transcription of *GSDME* by binding to the IRF1/RelA binding sites in the promoter region. Full-length amphioxus gasdermin E (*Bb*GSDME) can be cleaved by *Bb*CASP1/2/3-like at 2 different sites, D253 and D304, generating 2 distinct N-terminal fragments, p30 and p40. p40 can be further processed by *Bb*CASP1/2/3-like to form the pyroptotic p30. p30 translocates to the plasma membrane and forms pores, leading to pyroptosis. p40 adopts an autoinhibited conformation and can interact with p30 to inhibit *Bb*GSDME pore formation, providing a negative feedback mechanism for *Bb*GSDME-mediated pyroptosis. The figure was created in BioRender.

In their study, Wang and colleagues focused on the invertebrate amphioxus and found that amphioxus gasdermin E (*Bb*GSDME) induced significant pyroptosis in HeLa cells stimulated with TNFα and cycloheximide for the activation of caspase-3, and identified 3 caspase homologs (*Bb*CASP1/2/3-like) as the upstream enzymes of *Bb*GSDME [8]. Interestingly, *Bb*GSDME contains 2 caspase cleavage sites (at D253 and D304) responsible for generating a p30 or a p40 N-terminal fragment of *Bb*GSDME, respectively, upon cleavage by caspases. However, only the p30 fragment could induce pyroptosis in HeLa cells, whereas the p40 fragment exhibited no pyroptotic activity unless it was further processed by caspases to become p30. Using AlphaFold, the authors predicted an autoinhibited conformation of p40 mediated by the region spanning residues 254–304 in the C-terminus. The region of 254–304 binds to the N-terminal β1-β2 loop in p40, thereby preventing its attachment to the membrane but not affecting its self-oligomerization. Surprisingly, p40 functioned as a negative regulator of *Bb*GSDME-mediated pyroptosis by directly interacting with the p30 fragment, indicating a novel feedback mechanism fine-tuning gasdermin-mediated pyroptosis (Fig 1). Notably, expression of *Bb*GSDME is also alternative-splicing regulated, which may increase the complexity of regulating pyroptosis in amphioxus.

Wang and colleagues then identified several interferon regulatory factor 1 (IRF1) or RelA binding sites in the promoter region that control the expression of *Bb*GSDME (Fig 1). Transcription factors *Bb*IRF1 and *Bb*IRF8 are responsible for recognizing these regulatory elements and triggering the expression of *Bb*GSDME. It is noteworthy that IRFs primarily regulate immune response genes in response to pathogen invasion and are considered crucial mediators of pro-inflammatory responses. Previous studies have also identified IRF1/IRF2 binding sites in the promoter regions of mammalian *GSDMB* and *GSDMD* [11], further confirming the immune relevance of *Bb*GSDME in amphioxus and suggesting a conserved mechanism regulating the transcription of *GSDM* genes.

Wang and colleagues then generated an atomic model of *Bb*GSDME in the pore conformation based on the structure of human gasdermin D [8]. The simulated model, combined with their biochemical studies, revealed highly conserved lipid-binding and oligomerization interfaces in *Bb*GSDME. Interestingly, many single nucleotide polymorphisms (SNPs) of *GSDME* were identified that altered the pyroptotic function of gasdermin E, including the mutations K120Q or P212L. K120 is predicted to be a potential ubiquitination site, suggesting a possible mechanism of posttranslational modification to regulate human gasdermin E activity. Other *GSDM* genes, such as *GSDMB* and *GSDMD*, also exhibit SNPs, highlighting the importance of identifying functionally relevant SNPs in *GSDM* genes to better understand the regulation of gasdermin-mediated pyroptosis and its relevance to various diseases.

Gasdermins are associated with many diseases, including inflammatory bowel disease, asthma, Alzheimer’s disease, and cancers. In this study, Wang and colleagues found that *Bb*GSDME was involved in muscle necrosis in amphioxus upon bacterial infection. Specifically, infection by the bacteria *Edwardsiella tarda* caused significant tissue damage in the pharyngeal gill slits, skin, and intestines of amphioxus, which all express *Bb*GSDME. Treatment with Ac-VHTD-CHO, a *Bb*GSDME-D253 cleavage-specific inhibitor, significantly alleviated necrosis in these tissues.

Overall, the new study provides insights into the origin and evolution of the gasdermin family across metazoans [8]. The functional analysis of *Bb*GSDME in amphioxus highlighted its ancient role in innate immune response and revealed a novel negative feedback regulation of gasdermin-mediated pyroptosis. However, the regulation of the 2 cleavages of *Bb*GSDME in amphioxus and any potential preference of *Bb*CASPs for these cleavage sites remain unclear. Additionally, while human (and other mammalian) gasdermin E lacks the extra caspase-3 cleavage site, it is unknown whether this feedback regulation is unnecessary in these organisms or if alternative mechanisms exist. Given the significance of gasdermins in immune responses and disease, further research may uncover other regulatory mechanisms for their activation and inhibition.
